# Limitation of developmental test to measure functional independence of children: Relationship between the Japanese version of WeeFIM II® and KSPD

**DOI:** 10.3233/PRM-210079

**Published:** 2022-12-29

**Authors:** Mizuki Sugiyama, Sayaka Aoki, Nobuyuki Kawate, Keiji Hashimoto

**Affiliations:** a Department of Rehabilitation Medicine, Showa University School of Medicine, Yokohama, Japan; b Division of Ophthalmology, National Center for Child Health and Development, Tokyo, Japan; c Division of Orthopaedic Surgery, National Center for Child Health and Development, Tokyo, Japan; d Research Center for Advanced Science and Technology, The University of Tokyo, Tokyo, Japan

**Keywords:** Functional Independence Measure for Children (WeeFIM II®); Kyoto Scale of Psychological Development (KSPD), Activities of Daily Living (ADL), infants, toddlers

## Abstract

**PURPOSE:**

The purpose of the study was to explore whether a developmental test provides enough information to estimate a child’s functional independence. The strength of the relationship between developmental level and functional independence of different skills was investigated.

**METHODS:**

The participants were 397 children (age 0–7 years) who were referred to NCCHD for suspected developmental delay. The participants’ developmental level was measured with the Kyoto Scale of Psychological Development (KSPD) and their functional independence was assessed through a Japanese version of the WeeFIM II®. To calculate the strengths of the relationship between developmental age (DA) in different domains of the KSPD and WeeFIM II item scores, partial correlation analyses were conducted, controlling for chronological age.

**RESULTS:**

Partial correlation coefficients between the score of each of the 18 WeeFIM II items and each of the three KSPD domain DAs (controlling chronological age) fell in the ranges of *r* = 0.169–0.581 (Posture-Motor; P-M), *r* = 0.377–0.627 (Cognitive-Adaptive; C-A), and *r* = 0.332–0.655 (Language-Social; L-S). When the participants were divided into three age groups, the correlations ranged from –0.095 to 0.552 for the youngest group (mean age = 14.21 months), from 0.283 to 0.653 for the middle group (mean age = 32.98 months), and from 0.345–0.692 for the oldest group (mean age = 57.48 months), depending on the combinations of the WeeFIM II items/developmental domains of the KSPD. The results indicated that for most of the daily living skills, its functional independence was only partially explained by the scores of a developmental test, once chronological age was controlled.

**CONCLUSION:**

This study showed the limitation of a developmental test as a tool to measure the functional independence of children and the necessity of using an independent tool, such as WeeFIM II, to evaluate the level of required support for each daily living skill.

## 1 Introduction

In the field of disability and welfare services, it is important to measure a child’s level of functional independence and degree of required support to determine the amount of service provided for each individual. For adults with disability, Functional Independence of Measure (FIM) [1] has been often used for this purpose. FIM rates the level of functioning in different daily activities on a 7-point ordinal scale ranging from total dependence to complete independence and was found to be a valid and reliable measure for a diverse population [2–4].

The information related to the level of functional independence is also important to create appropriate service plans for children with disability. However, In Japanese rehabilitation settings, instead of gathering such information formally and systematically, the service providers are likely to rely too much on the results of a developmental test, such as the Kyoto Scale of Psychological Development (KSPD) [5] and Tanaka-Binet intelligence scale [6]. In other words, most service providers administer only a cognitive test or a developmental test to develop a service plan for children. While cognitive functioning is found to be associated with adaptive functioning, according to the results of meta-analysis, the strength of their relationship depends on an individual’s intelligence quotient [7], indicating the possibility that the developmental level of an individual is not straightforwardly related to the required support level. Additionally, it is possible that the developmental level is strongly related to the independence of some skills (e.g., bathing and locomotion), but not to other skills. These indicate that even though a developmental test itself is an important tool to determine the types and level of primary activities that are provided in educational and rehabilitation settings, it is unclear whether the administration of only a developmental test leads to gathering enough information to clarify the amount of support that is necessary and sufficient for each child. To examine this issue, it is necessary to figure out the strength of relationships between the scores of a developmental test and children’s functional independence.

In this study, to address the question above, the strength of the relationships between the developmental level of each domain measured by the KSPD and the degree of required support reflected in the item scores of the Functional Independence Measure for Children (WeeFIM II^®^) [8], a measure of functional independence, was investigated for children with various disorders or conditions who need to be evaluated to determine the types and amount of rehabilitation services they receive. Since chronological age is known to be related to both developmental level and level of functional independence, to reduce its influence on the relationships, partial correlation analyses were conducted.

## 2 Materials and methods

### 2.1 Overview

This study was conducted as a secondary analysis of the dataset collected between December 2011 and August 2014 at the National Center for Child Health and Development (NCCHD), Japan, as a part of the project related to the Japan Environment and Children’s Study entitled, “Investigation of the reliability and validity of the Japanese version of the Ages and Stages Questionnaires (J-ASQ).” For this project, to examine the validity of the J-ASQ, a variety of tools measuring children’s development and functioning were administered. In this particular study, the data from a subgroup of the participants who were administered the KSPD and WeeFIM II were used.

### 2.2 Participants

The participants of this study were 397 children (258 males, 139 females) aged 0–7 years who were referred to the Developmental Evaluation Center of the NCCHD due to risk or suspicion of developmental delay caused by various disorders or conditions. Among the participants, 97 (24.4%) were diagnosed with perinatal complications, 88 (22.2%) with a congenital disease, 86 (21.7%) with a central nervous system disease, 53 (13.4%) with a developmental disorder, 34 (8.6%) with an endocrine, nutritional, or metabolic disease, and 39 (9.8%) with other conditions.

For the purpose of analyses, these participants were divided into three subgroups. First, children aged four or older (oldest group; *n* = 83) were separated because KSPD does not always derive developmental age (DA) of the motor domain for those in this age range, while motor development of younger children can always be measured (see 2.3.1. for more detail). After separating this group, the rest of the participants were divided into half, those who were older than the median age (age 697 days = 1 year 10 months) of this subgroup (middle group, *n* = 158) and those who were younger (youngest group, *n* = 156). The descriptive information including sex, age, and disorders/chronic conditions of children in each group was presented in Table 1.

**Table 1:** Characteristics by the age group

	youngest group (<1 years 10 months) (Avg. 14.21 months) *n* = 156		middle group (Avg. 32.98 months) *n* = 158		oldest group ( $\underline{⩾}$ 4 years) (Avg. 57.48 months) *n* = 83	
Males/Females	109/47		102/56		47/36	
Age, mean(days) (SD)	447.67	(154.92)	1017.72	(208.53)	1737.59	(132.87)
Conditions, n (%)
Premature birth and low birth weight	43	(27.56%)	46	(29.11%)	8	(9.64%)
Congenital disease	46	(29.49%)	27	(17.09%)	15	(18.07%)
Central nervous system disease	36	(23.08%)	30	(18.99%)	20	(24.10%)
Developmental disorder	10	(6.41%)	25	(15.82%)	18	(21.69%)
Endocrine, nutritional, or metabolic disease	8	(5.13%)	16	(10.13%)	10	(12.05%)
Other conditions	13	(8.33%)	14	(8.86%)	12	(14.46%)

SD: standard deviation.

### 2.3 Measures

#### 2.3.1 Kyoto Scale for Psychological Development

The Kyoto Scale for Psychological Development (KSPD) (2001 version) [5] was used to measure the developmental level of the participants. This is a standardized developmental test that has been used widely in the clinical setting in Japan, specifically for young children. It is administered individually face-to-face and provides the examinee’s overall developmental age (DA) as well as total developmental quotient, which is calculated by dividing the estimated DA by chronological age. The test also yields DAs and developmental quotients in three distinct developmental domains: Postural-Motor (P-M), Cognitive-Adaptive (C-A), and Language-Social (L-S). It should be noted that due to the range of difficulty of the motor items in the KSPD, P-M DA cannot be calculated for children aged 4 or more if their motor development is within expectation. The KSPD has shown good split-half reliability on its domain scores [5] and good construct validity, with its developmental quotient score being highly correlated (*r* = 0.88) with the full-scale intelligence quotient obtained through the Tanaka-Binet Intelligence Scale (Japanese version of the Binet Intelligence Scale) [6, 9].

#### 2.3.2 Functional Independence Measure for Children, 2nd version

The Functional Independence Measure for Children, second version (WeeFIM II) system [8] was used to assess the degree of required support among the participants. This tool is an updated version of WeeFIM (Functional Independence Measure for Children) [10], which was developed to measure the functional independence of typically developing children aged from six months to seven years and those aged eight years or older who show delay or disability. WeeFIM II consists of the same 18 items as the original WeeFIM: eight Self-Care items (Eating, Grooming, Bathing, Dressing - Upper Body, Dressing - Lower Body, Toileting, Bladder Management, and Bowel Management), five mobility (Chair and Wheelchair, Toilet, Tub and Shower, Walk, Wheelchair, and Crawl, and Stairs), and five Cognition (Comprehension, Expression, Social Interaction, Problem Solving, and Memory). For many of the items in WeeFIM II, clearer descriptions are provided for rating purposes. Each of the items is rated on a 7-point ordinal scale ranging from total dependence (1) to complete independence (7), based on direct observations and interviews with caregivers.

While the reliability and validity of the WeeFIM II system have not been reported, the original and Japanese versions of WeeFIM were found to be reliable and valid [11–13].

### 2.4 Data collection procedure

Both WeeFIM II and the KSPD were administered and scored by three experts in developmental assessment, that is, two psychologists and a speech-language therapist who had sufficient experience in utilizing the testing materials and working with disabled infants and toddlers. During the data collection, one of the examiners administered KSPD to a participating child while another examiner interviewed their parents at the same time in a different room and collected the responses to the WeeFIM II. As indicated in the manual of the KSPD, when a participating child showed difficulty separating from their parent, the two tests were administered in the same room. In that case, the parent was asked to refrain from telling the answers to the questions by words or gestures.

### 2.5 Data analysis

To investigate the relationship between the level of development in different domains and the degree of support needed to carry out different daily tasks, correlations between the domain DAs of the KSPD and the total and item scores of WeeFIM II were calculated. Since chronological age is expected to be related to both developmental level and functional independence, to reduce its effect, partial correlation analyses were conducted, controlling for the chronological age of the participants. The calculations were first conducted for all of the participants, and then for each age subgroup. The size of the correlation was evaluated into four categories according to the rule of thumb for interpreting the size of the correlation coefficient [14] as follows; high correlation (–0.70 to –0.90), moderate correlation (–0.50 to –0.70), low correlation (–0.30 to –0.50), negligible correlation (–0.00 to –0.30). All statistical analyses were conducted using IBM SPSS Statistics Version 27.0. The significance level was set at α= 0.05 (two-tailed), and *p* < 0.05 was considered statistically significant. Due to the large number of correlation coefficients calculated, the *p*-values derived from the statistical analyses were adjusted using the Benjamini-Hochberg correction (1995), providing q-values in the Tables showing the results of correlation analyses.

## 3 Results

### 3.1 Partial correlation analyses for all the participants

The descriptive data of the participants, that is, the means and standard deviations (SDs) of the total developmental age (DA) and three domain DAs of the Kyoto Scale for Psychological Development (KSPD) and those of the total and item scores of WeeFIM II for each of the age subgroups are presented in Tables 2 and 3. For the readers who are interested in the means and SDs of the scores of these measures for different diagnostic groups, these were also calculated and can be found in Supplementary Tables 1–3.

**Table 2:** Descriptive data for the KSPD scores

	youngest group		middle group		oldest group	
	(<1 years 10 months)		(Avg. 32.98 months)		( $\underline{⩾}$ 4 years)	
	(Avg. 14.21 months)		*n* = 158		(Avg. 57.48 months)	
	*n* = 156			*n* = 83	
	Mean (days)	SD	Mean (days)	SD	Mean (days)	SD
Total DA	341.57	149.71	795.66	303.45	1266.71	517.94
Postural-motor DA	317.26	172.66	805.25	336.91	–	–
Cognitive-adaptive DA	353.74	156.70	815.67	316.26	1274.33	524.73
Language-social DA	342.55	139.63	771.94	315.65	1269.23	545.82

KSPD: Kyoto Scale of Psychological Development. SD: standard deviation. DA: developmental age.

**Table 3:** Descriptive data for the WeeFIM II scores

	youngest group		middle group		oldest group	
	(<1 years 10 months)		(Avg. 32.98 months)		( $\underline{⩾}$ 4 years)	
	(Avg. 14.21 months)		*n* = 158		(Avg. 57.48 months)	
	*n* = 156			*n* = 83	
	Mean	SD	Mean	SD	Mean	SD
Self-Care
Eating	1.93	0.82	3.04	1.17	3.92	1.42
Grooming	1.22	0.43	2.13	0.80	2.84	1.17
Bathing	1.18	0.40	1.84	0.80	2.55	1.23
Dressing-upper body	1.51	0.62	2.66	1.07	3.77	1.50
Dressing-lower body	1.44	0.65	3.03	1.28	3.98	1.50
Toileting	1.01	0.11	1.65	0.96	3.13	1.49
Bladder management	1.13	0.44	2.06	1.58	4.25	1.90
Bowel management	1.33	0.55	2.47	1.89	5.23	2.27
Mobility
Chair, wheelchair	1.67	1.20	3.72	1.50	4.73	1.70
Toilet	1.04	0.30	2.05	1.66	4.36	2.02
Bathtub, shower	1.51	0.84	3.12	1.22	4.24	1.52
Walk, wheelchair, crawl	2.81	1.32	4.22	1.01	4.82	0.98
Stairs	1.34	0.84	2.99	1.19	3.96	1.38
Cognition
Comprehension	1.67	0.59	2.59	0.97	3.23	1.24
Expression	1.45	0.56	2.61	1.12	3.18	1.26
Social interaction	1.28	0.46	2.01	0.77	2.67	1.07
Problem solving	1.33	0.47	2.08	0.68	2.60	0.91
Memory	1.54	0.57	2.51	1.06	3.47	1.24

SD: standard deviation. WeeFIM II: Functional Independence Measure for Children, 2nd version.

The partial correlations between domain DAs of the KSPD and the item scores of WeeFIM II are shown in Table 4. To interpret the results accurately, it should be noted that developmental ages in Cognitive-Adaptive domain (C-A DA) and Language- Social domain (L-S DA) were calculated for all the participants while developmental age in Postural- Motor domain (P-M DA) was calculated only for participants aged 3 or younger. The partial correlations between each of the three KSPD domain DAs and the score of each of the 18 WeeFIM II items fell in the ranges of *r* = 0.169–0.581 (P-M), *r* = 0.377–0.627 (C-A), and *r* = 0.332–0.655 (L-S).

**Table 4:** Partial correlations between the domain DAs of the KSPD and the WeeFIM II scores

KSPD domain DA
	Postural-motor	Cognitive-adaptive	Language-social
	DA (*n* = 314)	DA (*n* = 396)	DA (*n* = 396)
Self-Care		*p*	q		*p*	q		*p*	q
Eating	0.312	<0.00001	<0.001	0.473	<0.00001	<0.001	0.406	<0.00001	<0.001
Grooming	0.379	<0.00001	<0.001	0.581	<0.00001	<0.001	0.515	<0.00001	<0.001
Bathing	0.245	0.00001	<0.001	0.401	<0.00001	<0.001	0.366	<0.00001	<0.001
Dressing-upper body	0.349	<0.00001	<0.001	0.590	<0.00001	<0.001	0.526	<0.00001	<0.001
Dressing-lower body	0.444	<0.00001	<0.001	0.617	<0.00001	<0.001	0.562	<0.00001	<0.001
Toileting	0.181	0.00130	0.001	0.509	<0.00001	<0.001	0.525	<0.00001	<0.001
Bladder management	0.184	0.00110	0.001	0.503	<0.00001	<0.001	0.487	<0.00001	<0.001
Bowel management	0.169	0.00264	0.003	0.466	<0.00001	<0.001	0.461	<0.00001	<0.001
Mobility
Chair, wheelchair	0.511	<0.00001	<0.001	0.510	<0.00001	<0.001	0.444	<0.00001	<0.001
Toilet	0.174	0.00195	0.002	0.494	<0.00001	<0.001	0.477	<0.00001	<0.001
Bathtub, shower	0.471	<0.00001	<0.001	0.460	<0.00001	<0.001	0.405	<0.00001	<0.001
Walk, wheelchair, crawl	0.493	<0.00001	<0.001	0.377	<0.00001	<0.001	0.332	<0.00001	<0.001
Stairs	0.581	<0.00001	<0.001	0.511	<0.00001	<0.001	0.462	<0.00001	<0.001
Cognition
Comprehension	0.314	<0.00001	<0.001	0.575	<0.00001	<0.001	0.552	<0.00001	<0.001
Expression	0.332	<0.00001	<0.001	0.574	<0.00001	<0.001	0.594	<0.00001	<0.001
Social interaction	0.212	0.00016	0.000	0.544	<0.00001	<0.001	0.535	<0.00001	<0.001
Problem solving	0.271	<0.00001	<0.001	0.570	<0.00001	<0.001	0.566	<0.00001	<0.001
Memory	0.320	<0.00001	<0.001	0.627	<0.00001	<0.001	0.655	<0.00001	<0.001

Moderate correlation (–0.50 to –0.70); Low correlation (–0.30 to –0.50);             negligible correlation (–0.00 to –0.30). WeeFIM II: Functional Independence Measure for Children, 2nd version. KSPD: Kyoto Scale of Psychological Development. DA: developmental age.

### 3.2 Partial correlation analyses for different age groups

Next, to examine the age difference in relationships between the level of development in each domain and the required level of support for various tasks, partial correlations were computed between the domain-level DAs and WeeFIM II item scores for three different age groups: the youngest group (age <1 years 10 months), the middle group (age $\underline{⩾}$ 1 years 10 months and age <4 years), and the oldest group (age $\underline{⩾}$ 4 years). The results of the correlation analyses are displayed in Tables 5–7. The ranges of partial correlations between the subdomain DAs of the KSPD and the scores of each WeeFIM II item were –0.095–0.552 (P-M), –0.022–0.365 (C-A), and –0.041–0.416 (L-S) for the youngest group, 0.283–0.638 (P-M), 0.326–0.559 (C-A), and 0.342–0.653 (L-S) for the middle group, and 0.432–0.695 (C-A), and 0.345–0.692 (L-S) for the oldest group. The partial correlation between DA and WeeFIM II item scores in each domain increased with age.

For the youngest group, the results of partial correlation analyses between the KSPD domain DAs and WeeFIM II item scores indicated that a moderate correlation was found only for the relationship between P-M DA and Mobility: Stairs. The other WeeFIM Mobility items except for Mobility: Toilet as well as two dressing items (Dressing - Upper Body and Dressing - Lower Body) showed low correlations with P-M DA. Dressing - Lower Body, Comprehension, and Problem Solving showed low correlations with both C-A DA and L-S DA. The other WeeFIM II items that showed a low correlation with C-A DA were Grooming and Mobility: Chair, Wheelchair. Items that showed a low correlation with L-S DA were Expression and Memory. The overall results of the correlation analyses for this age group are presented in Table 5.

**Table 5:** Partial correlations between the domain DAs of the KSPD and the WeeFIM II scores for children in the youngest group (Age less than 1 year 10 months) (Mean age = 14.21 months)

WeeFIM II item(*n* = 156)	KSPD domain DA								
	Postural-motor			Cognitive-adaptive			Language-social
Self-Care	DA	*p*	q	DA	*p*	q	DA	*p*	q
Eating	0.181	0.02392	0.036	0.258	0.00117	0.003	0.167	0.03758	0.052
Grooming	0.282	0.00038	0.001	0.345	0.00001	<0.001	0.266	0.00082	0.002
Bathing	0.248	0.00183	0.004	0.286	0.00030	0.001	0.195	0.01491	0.024
Dressing-upper body	0.305	0.00011	<0.001	0.244	0.00226	0.005	0.240	0.00267	0.005
Dressing-lower body	0.473	<0.00001	<0.001	0.359	<0.00001	<0.001	0.416	<0.00001	<0.001
Toileting	–0.095	0.24108	0.296	–0.022	0.78419	0.830	–0.103	0.20030	0.258
Bladder management	–0.013	0.87366	0.874	0.098	0.22332	0.280	0.063	0.39466	0.474
Bowel management	–0.053	0.51287	0.589	–0.020	0.80629	0.837	–0.037	0.64624	0.712
Mobility
Chair, wheelchair	0.471	<0.00001	<0.001	0.365	<0.00001	<0.001	0.226	0.00473	0.009
Toilet	0.055	0.49461	0.581	0.037	0.64872	0.701	–0.041	0.60991	0.686
Bathtub, shower	0.479	<0.00001	<0.001	0.292	0.00022	<0.001	0.200	0.01275	0.021
Walk, wheelchair, crawl	0.455	<0.00001	<0.001	0.246	0.00201	0.004	0.229	0.00411	0.008
Stairs	0.552	<0.00001	<0.001	0.203	0.01117	0.019	0.180	0.02501	0.037
Cognition
Comprehension	0.185	0.02141	0.033	0.343	0.00001	<0.001	0.376	<0.00001	<0.001
Expression	0.144	0.07421	0.100	0.237	0.00297	0.006	0.385	<0.00001	<0.001
Social interaction	0.014	0.86125	0.877	0.248	0.00183	0.004	0.220	0.00595	0.010
Problem solving	0.119	0.13999	0.184	0.337	0.00002	<0.001	0.303	0.00013	<0.001
Memory	0.173	0.03145	0.045	0.285	0.00033	<.001	0.373	<0.00001	<0.001

Moderate correlation (–0.50 to –0.70); Low correlation (–0.30 to –0.50);             negligible correlation (–0.00 to –0.30). WeeFIM II: Functional Independence Measure for Children, 2nd version. KSPD: Kyoto Scale of Psychological Development. DA: developmental age.

For the middle group, the partial correlations between the WeeFIM II items and KSPD domain DAs were higher than those for the youngest group, for most of the combinations. P-M DA was moderately correlated with Mobility items except for Transfer: Toilet and showed a low correlation with items in other categories, except for Bathing. C-A DA and L-S DA were moderately associated with Dressing - Lower Body, Comprehension, Expression, and Memory. They were also found to have low correlations with all other items except for several items: Mobility: Stairs had a moderate correlation with C-A DA but a low correlation with L-S DA, while Social Interaction and Problem Solving showed a moderate correlation with L-S DA, but a low correlation with C-A DA. See Table 6 for the results of the correlation analyses for the middle group.

**Table 6:** Partial correlations between the domain DAs of the KSPD and the WeeFIM II scores for children in the middle group (Age between 1 year 10 months and 4 years) (Mean age = 32.98 months)

WeeFIM II item(*n* = 158)	KSPD domain DA								
	Postural-motor			Cognitive-adaptive			Language-social
Self-Care	DA	*p*	q	DA	*p*	q	DA	*p*	q
Eating	0.381	<0.00001	<0.001	0.438	<0.00001	<0.001	0.404	<0.00001	<0.001
Grooming	0.475	<0.00001	<0.001	0.475	<0.00001	<0.001	0.450	<0.00001	<0.001
Bathing	0.285	0.00030	<0.001	0.326	0.00003	<0.001	0.372	<0.00001	<0.001
Dressing-upper body	0.416	<0.00001	<0.001	0.442	<0.00001	<0.001	0.423	<0.00001	<0.001
Dressing-lower body	0.487	<0.00001	<0.001	0.537	<0.00001	<0.001	0.509	<0.00001	<0.001
Toileting	0.312	0.00007	<0.001	0.445	<0.00001	<0.001	0.491	<0.00001	<0.001
Bladder management	0.330	0.00002	<0.001	0.454	<0.00001	<0.001	0.402	<0.00001	<0.001
Bowel management	0.341	0.00001	<0.001	0.412	<0.00001	<0.001	0.417	<0.00001	<0.001
Mobility
Chair, wheelchair	0.580	<0.00001	<0.001	0.478	<0.00001	<0.001	0.405	<0.00001	<0.001
Toilet	0.283	0.00033	<0.001	0.372	<0.00001	<0.001	0.342	0.00001	<0.001
Bathtub, shower	0.513	<0.00001	<0.001	0.429	<0.00001	<0.001	0.386	<0.00001	<0.001
Walk, wheelchair, crawl	0.513	<0.00001	<0.001	0.476	<0.00001	<0.001	0.459	<0.00001	<0.001
Stairs	0.638	<0.00001	<0.001	0.510	<0.00001	<0.001	0.469	<0.00001	<0.001
Cognition
Comprehension	0.389	<0.00001	<0.001	0.515	<0.00001	<0.001	0.542	<0.00001	<0.001
Expression	0.453	<0.00001	<0.001	0.555	<0.00001	<0.001	0.653	<0.00001	<0.001
Social interaction	0.325	0.00003	<0.001	0.473	<0.00001	<0.001	0.503	<0.00001	<0.001
Problem solving	0.355	0.00001	<0.001	0.496	<0.00001	<0.001	0.577	<0.00001	<0.001
Memory	0.427	<0.00001	<0.001	0.559	<0.00001	<0.001	0.645	<0.00001	<0.001

Moderate correlation (–0.50 to –0.70); Low correlation (–0.30 to –0.50);             negligible correlation (–0.00 to –0.30). WeeFIM II: Functional Independence Measure for Children, 2nd version. KSPD: Kyoto Scale of Psychological Development. DA: developmental age.

For the oldest group, both C-A DA and L-S DA showed low correlations with Bathing, Mobility: Tub, Shower, and Walk, Wheelchair, Crawl. The item Eating showed a moderate correlation with C-A DA and a low correlation with L-S DA. The other WeeFIM II items showed moderate correlations with C-A DA and L-S DA. These results are presented in Table 7 in detail.

**Table 7:** Correlations between the domain DAs of the KSPD and the WeeFIM II scores for children in the oldest group (Age 4 years or older) (Mean age = 57.48 months)

WeeFIM II item(*n* = 83)	Cognitive-adaptive			Language-social
Self-Care	DA	*p*	q	DA	*p*	q
Eating	0.548	<0.00001	<0.001	0.464	0.00001	<0.001
Grooming	0.612	<0.00001	<0.001	0.530	<0.00001	<0.001
Bathing	0.423	0.00008	<0.001	0.345	0.00148	0
Dressing-upper body	0.689	<0.00001	<0.001	0.585	<0.00001	<0.001
Dressing-lower body	0.677	<0.00001	<0.001	0.603	<0.00001	<0.001
Toileting	0.570	<0.00001	<0.001	0.545	<0.00001	<0.001
Bladder management	0.651	<0.00001	<0.001	0.616	<0.00001	<0.001
Bowel management	0.569	<0.00001	<0.001	0.520	<0.00001	<0.001
Mobility
Chair, wheelchair	0.585	<0.00001	<0.001	0.552	<0.00001	<0.001
Toilet	0.654	<0.00001	<0.001	0.604	<0.00001	<0.001
Bathtub, shower	0.497	<0.00001	<0.001	0.452	0.00002	<0.001
Walk, wheelchair, crawl	0.432	0.00005	<0.001	0.399	0.00020	<0.001
Stairs	0.583	<0.00001	<0.001	0.537	<0.00001	<0.001
Cognition
Comprehension	0.604	<0.00001	<0.001	0.558	<0.00001	<0.001
Expression	0.592	<0.00001	<0.001	0.574	<0.00001	<0.001
Social interaction	0.578	<0.00001	<0.001	0.557	<0.00001	<0.001
Problem solving	0.633	<0.00001	<0.001	0.600	<0.00001	<0.001
Memory	0.695	<0.00001	<0.001	0.692	<0.00001	<0.001

Moderate correlation (–0.50 to –0.70); Low correlation (–0.30 to –0.50);             negligible correlation (–0.00 to –0.30). WeeFIM II: Functional Independence Measure for Children, 2nd version. KSPD: Kyoto Scale of Psychological Development. DA: developmental age.

## 4 Discussion

The purpose of this study was to examine the validity of current practice, and heavy reliance on the results of developmental tests, by investigating the strength of the relationship between the developmental level in different domains and the degree of support required for a child to carry out various daily tasks. For this purpose, partial correlation analyses were performed between the domain developmental ages (DAs) of the Kyoto Scale for Psychological Development (KSPD) and the scores of 18 WeeFIM II items. The analyses showed that the correlations fell within the low to moderate range for most of the combinations, which indicates that level of required support to execute different daily tasks is not fully explained by the scores obtained in the developmental test. This further led to an inference that the administration of only a developmental test, which is current practice, does not provide enough information to estimate the appropriate amount of support for a child.

When comparing the results obtained for the three age groups, it was found that the level of independence of most daily living activities was not highly associated with the developmental level measured by a developmental test among very young children (aged under two). This was not surprising because almost all of the children at this age are completely dependent on adults in almost all of the aspects of their daily life, even the most developmentally advanced children, as can be seen in the mean scores of the WeeFIM II items (see Table 3). For almost all of the activities, the relationship between the level of functional independence and the scores of the developmental test was stronger as the children became older. This indicates a possibility that a developmental test becomes more useful as a tool to estimate functional independence for older children. Nevertheless, the size of correlations varied depending on items, which is further discussed in the following paragraphs.

As for the WeeFIM II Self-care items that are not related to toileting behavior (Eating, Grooming, Bathing, Dressing - Upper Body, and Dressing - Lower Body), Dressing - Lower Body was likely to show a higher correlation with DA in each of the three developmental domains than other items for children in the youngest and the middle group. The result indicates that scores of developmental tests provide relatively more information to understand dressing skills than other skills for young children. This seems to be reasonable taking into account the fact that dressing requires multiple steps, including different types of movements, which necessitates several different motor skills and cognitive skills. In contrast, eating, grooming, and bathing consist of single tasks, and if one possesses a specific skill that enables them to perform the specific task well, support from others is unnecessary. Because motor development could not be measured for children aged four or older due to the lack of items in the KSPD, further studies are necessary to figure out which area of development corresponded most with different self-support skills.

The scores of the items related to toileting, including Toileting, excretion control (Bladder Management and Bowel Management), and Mobility (Toilet), showed almost no correlation with the DAs of the three developmental domains for children in the youngest group. However, they showed a low range of correlation for those in the middle group and a moderate correlation for those in the oldest group. These results suggest that the outcomes of developmental tests are somewhat useful to measure the level of required support for toileting activities around age 2–4. This argument is also supported by findings from several previous studies showing the relationship between developmental delay and urinary incontinence [15–18]. The negligible correlations found for the youngest age group seem to reflect the fact that toilet transfers and toileting are not typical skills of a child under 2. Because motor development could not be measured for older children, further study is necessary to find out which domain’s developmental level is most strongly related to toileting skills.

Mobility items (except for Toilet) showed relatively higher correlations with motor development than development in other areas and the relationship was relatively consistent across different age groups. The relationship was unknown for the oldest children due to the unavailability of Postural-Motor (P-M) DA for those at this age. While it seems clear that Mobility skills are best explained by motor development, again, further studies are necessary to gather more empirical evidence. Finally, Cognition items had higher correlations with Cognitive-Adaptive (C-A) DA and Language-Social (L-S) DA than P-M DA and the relationships became stronger for older children. These results are within the expectation because many KSPD items measuring C-A DA and L-S DA consist of tasks that require the examinee to understand and comply with the direction, provide responses verbally, solve the given problems, and/or retain some information in their mind. Thus, it can be concluded that the degree of required support level for the skills in Communication and Social-cognitive areas is partially explained by the level of cognitive and language development measured by the KSPD, but not fully.

This study had several limitations. First, the participants were restricted to children who visited the Developmental Evaluation Center of the National Center for Child Health and Development due to risk or suspicion of developmental delay caused by various disorders or conditions. While the participants were a subset of the primary target population of WeeFIM II, children who are likely to need substantial assistance to complete daily tasks for various reasons, it is unclear if the results of this study are generalizable to other populations who are expected to need less support, for example, typically developing children. In addition, due to the cross-sectional feature of this study, it is unclear whether the age difference found in this study is generalizable. Second, because the KSPD does not include many motor items for older children and, as a result, P-M DA could not be obtained for the children aged four or older, the relationship between the WeeFIM II scores and motor development could not be examined for children in this age range. Third, as WeeFIM II measures the degree of support required to complete different tasks independently, it does not provide information about the “overall” level of support needed for an individual or their overall level of acquisition of daily living skills.

In conclusion, the results of this study indicate that the information obtained through a developmental test, KSPD, about the degree of required support for daily living skills is somewhat limited. This means that to determine the number of support resources allocated to each individual with a disability, it is important to directly measure the level of independence, not just rely on the results of the developmental test too much. For this purpose, while the updated version has not been validated yet, WeeFIM II can be recognized as a useful tool. To increase understanding of the psychometric characteristics of the tool, an independent validation study should be conducted for the WeeFIM II in future.

## 5 Conclusion

This study showed a low to moderate relationship between the degree of support required for a child to complete various daily tasks and their level of development in different domains, controlling for chronological age. The results indicate a limitation of developmental tests and the importance of using an appropriate tool to measure an individual’s required support level and create an effective intervention/accommodation plan.

## Supplementary Material

Supplementary Material

## References

[1] Uniform Data System for Medical Rehabilitation. The FIM ® Instrument: Its Background, Structure, and Usefulness. Buffalo: UDSMR; 2012.

[2] Kidd D , Stewart G , Baldry J , et al., The Functional Independence Measure: A comparative validity and reliability study, Disabil Rehabil 1995;17(1):10–4. doi: 10.3109/09638289509166622. 7858276

[3] Weiner BJ , Lewis CC , Stanick C , et al., Psychometric assessment of three newly developed implementation outcome measures, Implement Sci. 2017;12(1):108. doi: 10.1186/s13012-017-0635-3. 28851459 PMC5576104

[4] Pollak N , Rheault W , Stoecker JL , Reliability and validity of the FIM for persons aged 80 years and above from a multilevel continuing care retirement community, Arch Phys Med Rehabil 1996;77(10):1056–61. doi: 10.1016/s0003-9993(96)90068-4. 8857886

[5] Society for the Kyoto Scale of Psychological Development. TheKyoto Scale of PsychologicalDevelopment 2001: Information for standardization and administration. Kyoto: Kyoto Kokusai Shakai Fukushi Center; 2002 [in Japanese].

[6] Tanaka Institute for Educational Research. Tanaka–Binet Intelligence Scale V. Tokyo: Taken Publishing; 2003 [in Japanese].

[7] Alexander RM The Relation between Intelligence and Adaptive Behavior: A Meta-Analysis [dissertation]. Lawrence (KS): University of Kansas; 2017.

[8] Uniform Data System for Medical Rehabilitation. The WeeFIM II ® Clinical Guide. Version 6.4. Buffalo: UDSMR; 2016.

[9] Koyama T , Osada H , Tsujii H , Kurita H , Utility of the Kyoto Scale of Psychological Development in cognitive assessment of children with pervasive developmental disorders, Psychiatry Clin Neurosci 2009;63(2):241–3. doi: 10.1111/j.1440-1819.2009.01931.x. 19335396

[10] Msall ME , DiGaudio K , Rogers BT , et al., The Functional Independence Measure for Children (WeeFIM), Conceptual basis and pilot use in children with developmental disabilities. Clin Pediatr (Phila) 1994;33(7):421–30. doi: 10.1177/000992289403300708. 7525140

[11] Ottenbacher KJ , Taylor ET , Msall ME , et al., The stability and equivalence reliability of the functional independence measure for children (WeeFIM), Dev Med Child Neurol 1996;38(10):907–16. doi: 10.1111/j.1469-8749.1996.tb15047.x. 8870612

[12] Ziviani J , Ottenbacher KJ , Shephard K , Foreman S , Astbury W , Ireland P , Concurrent validity of the Functional Independence Measure for Children (WeeFIM) and the Pediatric Evaluation of Disabilities Inventory in children with developmental disabilities and acquired brain injuries, Phys Occup Ther Pediatr 2001;21(2):91–101. 12029858

[13] Liu M , Toikawa H , Seki M , Domen K , Chino N , Functional Independence Measure for Children (WeeFIM): A preliminary study in nondisabled Japanese children, Am J Phys Med Rehabil 1998;77(1):36–44. doi: 10.1097/00002060-199801000-00006. 9482377

[14] Mukaka MM . Statistics Corner: A guide to appropriate use of Correlation coefficient in medical research, Malawi Med J 2012;24(3):69–71. 23638278 PMC3576830

[15] Joinson C , Grzeda MT , von Gontard A , Heron J , A prospective cohort study of biopsychosocial factors associated with childhood urinary incontinence, Eur Child Adolesc Psychiatry 2019;28(1):123–30. doi: 10.1007/s00787-018-1193-1. 29980842 PMC6349792

[16] Kuniki T , Evacuation and urination of children with developmental disabilities [in Japanese], Hokkaido J Spec Needs Educ Emot Dev Disturb Pers 1986;5:89–92.

[17] Heron J , Grzeda MT , von Gontard A , Wright A , Joinson C , Trajectories of urinary incontinence in childhood and bladder and bowel symptoms in adolescence: Prospective cohort study, BMJ Open. 2017;7(3):e014238. doi: 10.1136/bmjopen-2016-014238. PMC5353296 28292756

[18] Hjälmås K Urinary incontinence in children: Suggestions for definitions and terminology. Scand J Urol Nephrol Suppl. 1992;141:1–6;discussion 18-9. 1609244

