# Supplementary material for: Limitation of developmental test to measure functional independence of children: Relationship between the Japanese version of WeeFIM II® and KSPD
Source: J Pediatr Rehabil Med. 2022 Dec 29;15(4):667–76. doi: 10.3233/PRM-210079 (PMC9881016; doi:10.3233/PRM-210079)
Supplement: Supplementary Material [file prm-15-prm210079-s001.docx]

**Supplementary table 1: Comparison of WeeFIM scores and KSPD DA for each 6 disease groups in youngest group (Age less than 1 year 10 month) (Mean age=14.21 months)**

KSPD: Kyoto Scale of Psychological Development

SD: standard deviation

DA: developmental age

**Supplementary table 2: Comparison of WeeFIM scores and KSPD DA for each 6 disease groups in middle group (Age between 1 year 10 month and 4 years) (Mean age=32.98 months)**

KSPD: Kyoto Scale of Psychological Development

SD: standard deviation

DA: developmental age

**Supplementary table 3: Comparison of WeeFIM scores and KSPD DA for each 6 disease groups in oldest group (Age 4 years or older) (Mean age=57.48 months)**

KSPD: Kyoto Scale of Psychological Development

SD: standard deviation

DA: developmental age
